# PD-L1, a Master Regulator of Immunity 2.0

**DOI:** 10.3390/ijms24054385

**Published:** 2023-02-23

**Authors:** Grazyna Kochan

**Affiliations:** Oncoimmunology Group, Navarrabiomed, Fundación Miguel Servet-Complejo Hospitalario de Navarra-UPNA-IdISNA, 31008 Pamplona, Spain; grazyna.kochan@navarra.es

Since the introduction of the first anticancer treatments at the beginning of the 20th century, many different chemotherapeutics have been developed [1]. The use of all these treatments has increased the overall survival of cancer patients. However, long-term survival (over five years) has, in general terms, been very low. A significant improvement in survival was achieved after the introduction of therapies targeted towards specific tumour mutations. Despite their fewer side effects and higher efficacies, the development of drug resistance is a major drawback [2]. The identification of immune checkpoint molecules on the surface of immune and cancer cells led to the development of therapies that greatly improved the overall survival of patients with numerous tumours. These highly promising results led to rapid progress in the development of different therapeutics blocking the immune checkpoints [3,4]. 

These novel immunotherapy treatments significantly extended overall survival and progression-free survival in treated individuals. However, a large group of patients still do not respond to these immunotherapies. The identification of all factors affecting therapy success is key. This entails the unveiling of the molecular mechanisms of intracellular signalling of these immune checkpoint molecules in health and disease. 

Different monoclonal antibodies targeting CTLA-4, PD-1, and LAG-3 are already applied in human therapy [5], and some of the most-used are monoclonal antibodies that block interactions between PD-L1 and PD-1 [6,7,8]. 

PD-L1 is of special interest as its presence on the cancer cell surface was demonstrated early on to be associated with the clinical success of immune checkpoint blockades [9]. In fact, the application of pembrolizumab (humanised IgG4 anti-PD-1 by Merck) is conditioned by the presence of PD-L1 on the surface of cancer cells. PD-L1 is a type I transmembrane protein of the B7 family of co-stimulatory and co-inhibitory molecules that play a central role in regulating systemic tolerance [7]. Its natural receptor, PD-1, is expressed on the surface of activated T, B, and NK cells [6]. PD-L1 engagement with PD-1 regulates the degree of activation of effector immune cells [10]. However, cancer cells up-regulate PD-L1 to avoid T cell attack [11,12,13]. In addition, PD-L1 confers survival advantages to the cancer cell by counteracting apoptosis mediated by interferons and many other pro-inflammatory mediators [14,15].

PD-L1 expression in cancer cells is currently used as a biomarker of response. However, we have to remember that PD-L1 expression on the cell surface is dynamic. Moreover, the histochemical determination of PD-L1 presence on the tumour cells in histopathological analyses can vary depending on the reagents used for analysis, sample preparation (fixation), or tumour fragment representation in a sample [16,17]. Additionally, it has been shown that immune responses triggered by immunotherapy treatments depend not only on PD-L1 expression levels on cancer cells’ surface, but also on the surface of myeloid compartment cells [18,19,20,21]. We have to be aware that myeloid cells are key regulators of T cell responses as well. It has also been previously shown that the degree of PD-L1 surface expression on myeloid cells can be associated with clinical responses to treatment. Therefore, a “PD-L1-negative tumour” can still respond to a PD-L1/PD-1 blockade by acting over the myeloid compartment [18]. Indeed, this molecule is also present as a soluble form in plasma from patients, where it likely plays a regulatory role as well.

Based on the “knowns and unknowns”, PD-L1 is a very attractive subject for research and a therapeutic target still undergoing exploitation. 

Following on from all clinical observations and basic investigations, more and more information is being gathered on the role of PD-L1. The current issue tries to contribute knowledge of PD-L1 in different tumours and autoimmune diseases, but also in physiological conditions. Until now, the majority of research has been centred mainly in the field of oncology. However, in the current issue we have some examples of the importance of PD-L1 expression and function in other aspects of human health. It is important to characterize PD-L1 expression and its role in different cancers and diseases, but also in physiological conditions. Gathering all this information together will help us to find analogies and similarities between cancers and other disorders. Only systematically filling the gaps in knowledge of the molecular mechanisms of action and the status quo of PD-L1 expression in health and disease will help us develop personalised treatments, not only for cancer, but also for other inflammatory disorders.
